# Nanomaterials for Tissue Engineering In Dentistry

**DOI:** 10.3390/nano6070134

**Published:** 2016-07-21

**Authors:** Manila Chieruzzi, Stefano Pagano, Silvia Moretti, Roberto Pinna, Egle Milia, Luigi Torre, Stefano Eramo

**Affiliations:** 1 Department of Civil and Environmental Engineering—UdR INSTM—University of Perugia, Strada di Pentima, 4–05100 Terni, Italy; luigi.torre@unipg.it; 2 Department of Surgical and Biomedical Sciences—University of Perugia, S. Andrea delle Fratte, 06156 Perugia, Italy; stefano.eramo@unipg.it; 3 Department of Experimental Medicine—University of Perugia Polo Unico Sant’Andrea delle Fratte, 06132 Perugia, Italy; silvia.moretti@unipg.it; 4 Department of Biomedical Science—University of Sassari viale San Pietro 43/C -07100 Sassari, Italy; rpinna@uniss.it (R.P.); emilia@uniss.it (E.M.)

**Keywords:** nanomaterials tissue engineering, dentistry, stem cells, signalling molecules, scaffolds

## Abstract

The tissue engineering (TE) of dental oral tissue is facing significant changes in clinical treatments in dentistry. TE is based on a stem cell, signaling molecule, and scaffold triad that must be known and calibrated with attention to specific sectors in dentistry. This review article shows a summary of micro- and nanomorphological characteristics of dental tissues, of stem cells available in the oral region, of signaling molecules usable in TE, and of scaffolds available to guide partial or total reconstruction of hard, soft, periodontal, and bone tissues. Some scaffoldless techniques used in TE are also presented. Then actual and future roles of nanotechnologies about TE in dentistry are presented.

## 1. Introduction

The application in dentistry of nanomaterials (NM) and tissue engineering (TE) have revolutionized perspectives and are changing their clinical activities. Since the beginning of the century, the possibilities of substitution and restoration of oro-maxillo-facial lost tissues for traumas or other pathologies were transplantation of graft or fabric autologous tissues, heterologous materials for bony or mucous losses, or with heterologous biocompatible materials (amalgam, composite resins, glass ionomer cements, guttapercha) in the partial loss of dental tissues or, additionally, the use of osteointegrable materials in implant dentistry for the complete replacement of teeth lost in toto [1].

NM were used in dentistry for the first time in 2002 [2,3,4] with the inclusion of nanofillers in composite resins for dental reconstruction. However, previously, the birth of nanodentistry [5,6] was already foreseen. Since then, some NM have been used in the improvement of restorative dentistry materials [7,8]:

- prevention of main oral and dental biofilm-dependent diseases, like caries and periodontal diseases, with the addition of antibacterial and antidemineralizing particles in toothpastes, mouthwashes, and composite resins [4,9,10,11,12,13], or of active nanoparticles for remineralization in toothpastes [14,15], composite resins, and dental adhesives [16,17];
- help in diagnosis of malignant and pre-malignant oral diseases with some means, such as contrast particles for CT imaging, like gold nanoparticles (GNPs) [18,19,20]; ”quantum dots”, semi-conductor crystals of nanoscale inserted in diseased tissues that behave like fluorophores when exposed to luminescence NIR (near-infrared) [21]; the “oral fluid nanosensor tests” (OFNASET) for identification of tumoral salivary biomarkers [22]. These last ones are also used in periodontal disease diagnosis, for their capability to identify specific periodontopatogenic bacteria [23], such as “electronic microchip-assays” able to detect C-reactive protein (CRP), a biomarker of the inflammation connected to periodontal disease [24];
- development of nano-textured surface formation in implantology [25,26,27,28,29,30,31].

Regarding TE in dentistry, the introduction of new membranes able to enhance guided tissue regeneration (GTR) in periodontology [32,33,34] and of autologous platelet concentrates (PRP and PRF) with membranes for guided restoration of bone loss in oral surgery, happened in 1980–1990 [35,36,37] and can be considered the beginning of the new techniques based on three principal elements of tissue engineering: stem cells, scaffolds, and signaling molecules [38].

In recent decades NM and TE have become integrated in dentistry with the introduction of nanotechnologies in the constitution of scaffold matrices (rigid and soft), the use of growth factors and stem cells, and with the introduction of biomodulation techniques for dental tissue reconstruction.

After the presentation of a short summary of the histological structure, the characteristics and the diseases of oral tissues, the aim of this review is to illustrate the dental and periodontal stem cells, the signaling molecules usable for scaffolds or in alternative scaffoldless techniques and, finally, the current knowledge strictly regarding the results of NM applications for TE in dentistry.

## 2. Structure and Diseases of Dental and Periodontal Tissues

**Figure 1:**
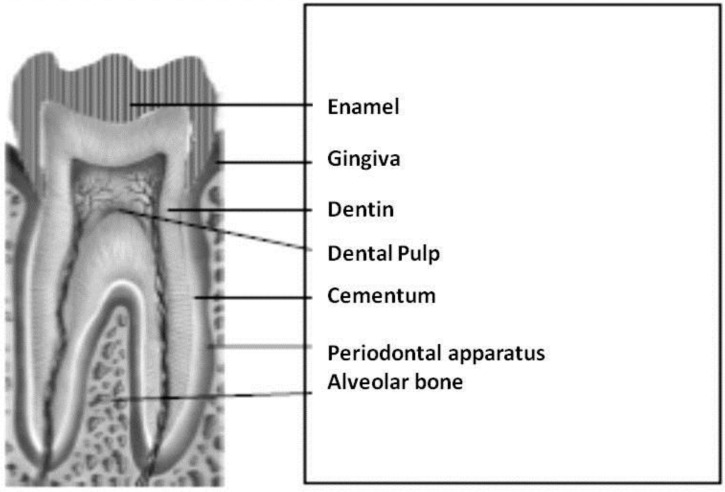
Schematic section of a tooth and surrounding tissues.

From an histological point of view, teeth are made of three strong tissues (enamel, dentin, and cementum) and dental pulp, in the middle, responsible for the trophism of the dentin [39] (Figure 1). The three hard tissues have a similar composition of inorganic components but with different percentages because enamel is fundamentally inorganic, while dentin and cement have an important organic component, and dental pulp is only organic.

From an embryological point of view, enamel has an epithelial–ectodermal origin, but its forming cells (ameloblasts), once completed their function, undergo apoptosis and they are not present in adult tissue (except for some epithelial cells in periodontal tissues called epithelialrests of Malassez-ERM). Instead, dentin and dental pulp (which together form the pulpodentinal complex) and the periodontal apparatus (made of cement, periodontal ligament (PDL), and alveolar bone) are of mesenchymal origin. Chemical composition and some functional parameters of the inorganic phases of the human calcified tissues [40] are shown in Table 1.

Enamel is the hardest tissue of the human body, it is acellular and translucent and forms the external surface of the teeth. It has different thicknesses, maximum in the cusps and minimum in the cementoenamel junction. It is made of polygonal prisms with a diameter of 2–3 microns (microstructure) and these are made of small hexagonal crystals of nanometric dimensions (nanostructure). The enamel has an inorganic component of 97% in weight, while the organic components are given by water (1.5% in weight) and proteic organic components (1.5% weight). The enamel organic component is made of small soluble peptides and insoluble proteic components (amelogenin, enamelin, ameloblastin) that presumably represent the residual component of the formative matrix tissue [41]. The inorganic component of the mature enamel is histologically organized in prisms (or rods) and these are made of hexagonal apatite crystals with an average thickness of 90 nm, a width of 30 nm, and a length of 100 nm [4,42]. Density and mineral content of crystals and prisms are uneven and decrease from the enamel surface to the dentin-enamel junction (DEJ) [43].

The composition of the crystallites is given by non-stoichiometric hydroxyapatite (HA) with ionic substitutions (Na^+^, K^+^, Mg^2+^, Sr^2+^ per Ca^2+^, carbonate instead of phosphate, fluoride, chloride, and carbonates instead of hydroxide) which is called “biological apatite” or dahllite, the true organic component of the tooth, bone, and pathological calcifications of human and animal bodies [44]. The average composition of the HA of the enamel was calculated in [(Ca)_8.68_(HPO_4_)_0.61_(CO_3_)_0.54_(PO_4_)_5.26_(OH)_0.1_] [45] and its solubility product (3.04 × 10^−59^) is higher than that of stoichiometric HA (then enamel is more soluble) [46], with values from 7.2 × 10^−53^ to 6.4 × 10^−58^ [47]. Crystal formation occurs through the cellular guide of ameloblasts (destined to disappear at the end of their activity) and it is given by an initial, epitaxial nucleation (guided by the primitive dentin formation) and then by primary and secondary crystalline growth regulated by the Tomes' processes of ameloblasts.

The crystallites are organized into prisms, or rods (constituted from about 1000 crystallites each) and oriented with the main crystallographic axis parallel to the major axis of the prism; the crystal’s columns have regular “cross-striations”, a sign of crystal growth. The prisms, with a diameter of 2–3 microns, are between 30 and 40 thousand per mm^2^.

**Figure 2:**
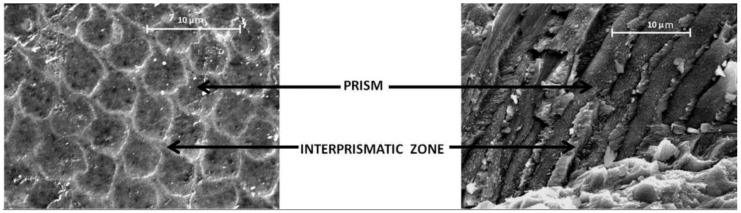
Prisms of the enamel observed on the surface and in cross-section.

On the border of every prism there is a different crystal orientation that provides an interface with a greater intercrystalline space (Figure 2) and where there is the higher quantity of water and organic components.

**Figure 3:**
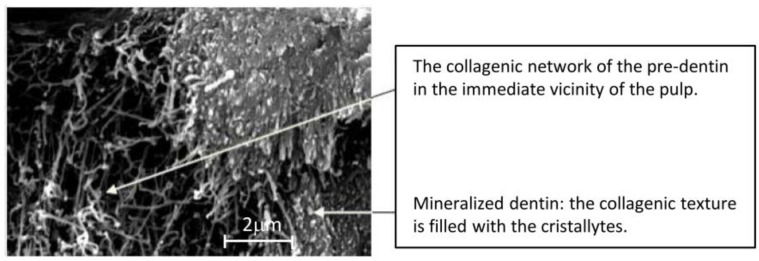
Dentin morphology with collagen fibers and inorganic components.

The dentin, covered by enamel in the crown and by cement in the root, is the main structure of the tooth. It is formed by the action of highly specialized cells, the odontoblasts, which, throughout the life of the tooth, continue to create new dentin. The nanostructure of the dentin is made by collagen fibers organized in a network in which the spaces are filled by crystals of biological hydroxyapatite with higher substitutions than in the enamel (Figure 3). Their dimensions are of an order of magnitude smaller than those of enamel and determine the hard-elastic consistency of the tissue.

**Figure 4:**
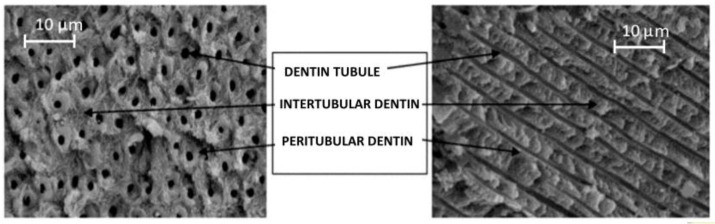
Dentinal tubules observed on the surface and in cross section.

The microstructure of the dentin is characterized by the presence of dentinal tubules (from 20,000 to 30,000 per mm^2^) with a diameter of 2 microns near the pulp and 1 micron at the enamel-dentin junction (EDJ) (Figure 4). They have Tomes’ cytoplasmatic extensions of the odontoblasts of which cell bodies are located in the outer portion of the pulp. The presence of the tubules allows recognition of a peritubular dentin (around the tubule) and intertubular dentin (between tubules).

Dentin has a composition with an inorganic portion (70%) of hydroxyapatite (HA) imperfectly crystallized non-stoichiometrically, with replacements of sodium carbonate or magnesium, and an organic portion (20) represented by type II collagen fibers and water (10%).

**Figure 5:**
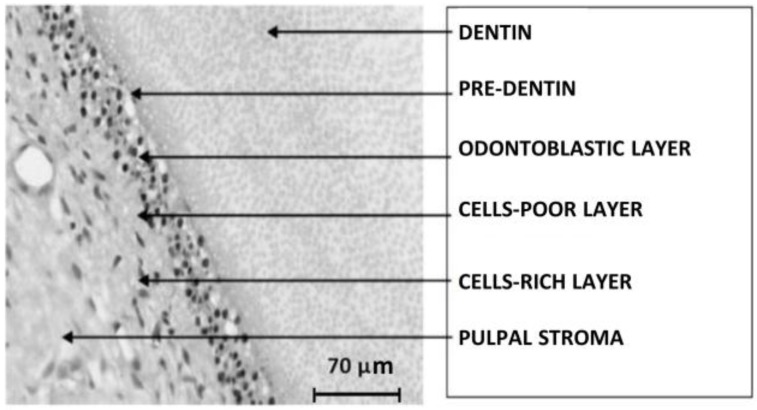
The structure of the dental pulp.

The dental pulp, contained in the pulpal cavity, is a loose connective tissue, of mesenchymal origin and it consists of hyaline substance, cells (odontoblasts, fusiform and stellar fibroblasts, macrophages), collagen fibers (in higher density in the oldest pulp), vessels, and nerves. From outside to inside, the pulp presents subsequent layers: pre-dentin, the odontoblasts layer, the poor cells layer, the rich cells layer, and the stroma (with vessels, nerves, and collagen fibers). Figure 5 shows the optical microscope image of the dental pulp structure.

The cementum is a hard tissue similar to bone that covers the tooth root and it is divided into two types: acellular cementum that covers the upper third of the root, and cellular cementum. Its thickness (10 micron at the enamel-cementum junction) increases towards the apex of the root (from 100 to 200 micron). In the coronal portion some cells are present (cementoblasts and cementocytes) and they are hosted in gaps similar to those of the bones. The main function of the cementum is to be the dental attachment point of periodontal ligament fibers.

The PDL is a very elastic connective cloth constituted of several groups of collagen fibers with an origin in alveolar bone (Sharpey’s fibers) and insertion in the cementum and on the alveolus walls. The groups of fibers have a different orientation and are distinct in: “Koelliker's annular ligament” from the ECJ to the marginal perimeter of the alveolus; the horizontal group presents in the coronal third of the PDL, which passes perpendicularly to the long axis of the tooth, under the annular ligament; the oblique group, in the middle third, which passes obliquely; the apical group which passes from the tooth apex to the base of the alveolus; and the interradicular group, present between root surfaces of the posterior teeth. The fibers are immersed in a fundamental substance (constituted of water, glycosaminoglycanes, and glycoproteins) and, between them, cells of several types are present, like fibroblasts (arranged parallel to the collagen component), macrophages, mesenchimal cells, undifferentiated cells, and ERM.

The alveolar bone constitutes the insertion surface of PDL fibers on the slope of the basal mandibular and maxillary bone. The alveolus walls are constituted by a thin layer of compact bone, while the space between the alveoli are formed by cancellous bone. The alveolar bone plays an important action of support and it is continually renewed in response to functional forces.

The main diseases of dental interest that may affect dental elements and their support apparatus are:

- damage to the hard tissues of tooth like caries, fractures, cervical erosions, without loss of pulpal functionality [48];
- damage to the pulpodentinal complex with loss of pulpar vitality and, consequently, with removal by endodontic therapy [49,50];
- damage to periodontal complex and alveolar bone from periodontal disease and trauma;
- complete loss of one or more teeth in the most severe forms of these diseases or their absence for agenesis;
- small or medium bone losses by mandibular or maxillary cysts or odontogenic tumors.

Currently, none of these pathological situations are resolved clinically with tissue engineering methods, but the research in vitro and in vivo is arriving at this type of application. Now, the methods that will represent the future aspects of research (stem cells, signaling molecules, biomaterials for scaffolds, and nanomaterials technologies) are presented.

## 3. Stem Cells in Dental and Periodontal Tissues Usable in Dentistry

Stem cells of non-dental origin, like those from bone marrow, adipose tissue, and induced pluripotent stem cells, were used to reconstruct dental tissues [51,52,53,54,55,56,57,58,59,60,61] and they may be useful as an alternative in the absence of teeth. Those of dental and periodontal origin are more promising due to their affinity with target tissues. They have been classified in [1,62,63]:

1. Pluripotent cells—called DPPSC (dental pulp pluripotent stem cells), isolated in third molars pulp [64], are potentially useful for regeneration of dental tissues both epithelial (enamel) that are mesenchymal;
2. Mesenchymal cells, isolated from the adult pulp (dental pulp stem cells or DPSC) [65] and from the deciduous exfoliated teeth (SHED) [66]; from the apical part of dental papilla (stem cells from apical papilla, or SCAP) [67,68]; from the dental follicle (dental follicle stem cells, or DFSC [69]; or from the PDL (PDLSC) [70];
3. Epithelial cells: although epithelial stem cells have been isolated in third molars [71] and in deciduous pulp [72], the most secure source is given by epithelial rests of Malassez-ERM [73,74].

## 4. Main Usable Growth Factors and Signaling Molecules

The use of biological mediators to induce or stimulate cell growth is present throughout tissue engineering applied to dentistry [75] but, in some sectors of dentistry, the application of growth factors had particular consideration. For example, growth factors and biological studies for periodontal regeneration have included bone morphogenetic proteins 2, 3, 4, 6, 7, 12; cell-binding peptide p-15; fibroblast growth factor-2; growth differentiation factor −5; insulin-like growth factor-1; matrix factors (fibronectin, amelogenins, thrombospondin); platelet-derived growth factor; platelet-rich plasma; vascular endothelial growth factor; and enamel matrix derivative [76,77,78]. In the case of pulpodentin complex, inductive growth may come from the regeneration of degraded dentin matrix [79], from revitalization process or synthesized in vitro and delivered into tissues [80]. Among them it is worth mention [81] recombinant human bone morphogenetic proteins-2 and -4 or rh BMP2 and rh BMP4 [82,83]; stromal cell derived factor (SDF)-1, basic fibroblast growth factor (BFGF) [84,85,86], platelet derived growth factor (PDGF), stem cells factor (SCF), granulocyte colony-stimulating factor (G-CSF), and VEGF proangiogenic factor [87,88].

## 5. The Main Biomaterials Usable to Build Scaffolds/Matrices

Generally, a scaffold, to serve as a suitable matrix to the reconstruction of tissue, should exhibit some important features: ease of handling, adequate porosity, pore shape and scale to permit penetration and diffusion of cells, growth factors, nutrients and easy removal of byproducts from the cells, biodegradability without release of toxin or harmful products, positive bioactivity, low immunogenicity, ability to allow the vascularization, and good physical and mechanical properties [89,90]. Regarding the mechanical behavior, the scaffolds in dentistry have an important distinction. In the bone, TE requires a rigid scaffold that reproduces the size and architecture of the tissue to be rebuilt; in the pulpodentinal complex and in the periodontal apparatus of the TE, due to the small size and difficulty to reach the receiving site, currently involves soft and injectable scaffolds. For this reason, the biomaterials used in scaffold formation can be classified according to the natural and synthetic sources, or depending on the physical consistency either rigid or soft. It is useful to remember that until a short time ago some techniques that used soft scaffolds (hydrogels) were wrongly defined “scaffoldless”.

The biomaterials used in the construction of rigid scaffolds have prevailing interest in surgery and are intended primarily to guide the rebuilding of bone and cartilage. They can be classified into natural or synthetic ceramics, natural or synthetic polymers, or composites/hybrids from these materials [38,91]. Naturally-derived ceramic materials include, in addition to autografts and allografts, bones [92,93], starch-based compounds [94], and coral derivatives [95]. Synthetic ceramics include inorganic materials, such as calcium phosphates (CaPs), bioactive glasses, and glass-ceramics [90,96]. Commonly used CaPs are monocalcium phosphate monohydrate, monocalcium phosphate anhydrous, dicalcium phosphate dihydrate, dicalcium phosphate anhydrous, octacalcium phosphate, b-tricalcium phosphate (TCP), amorphous CaP (ACP), calcium-deficient hydroxyapatite, and hydroxyapatite [97]; all of these are usually used in a mixed fashion to form cements (CPCs) usable as self-setting synthetic bone graft materials [98,99,100,101,102,103]. While most of the natural polymers are used mainly in soft matrices, there are different synthetic polymers usable to constitute rigid scaffolds, like polyhydroxylacids, poly-hydroxyalkenoates (PHAs), poly-hydroxybutyrates, and poly-propylene fumarates [104].

In particular the polyhydroxyl acids (the polyactic acid (PLA), the polyglycolic acid (PGA), the polylactideCo-glycolide (PLGA), and the poly-caprolactone (PCL) are the most studied because their structure, biodegradability, and substance release can be easily calibrated. Biomaterials used in the construction of soft scaffolds or hydrogel are intended primarily to guide the reconstruction of tissues of pulpodentinal complex and of periodontal apparatus given the special conditions in which they are located [51]. In vivo forming hydrogel can be prepared as a solution which gels, it can incorporate stem cells and signaling molecules simply by mixing, it can also reach the site through injection by syringe in vivo. They can be prepared by various biomaterials classified as natural, such as collagen, fibrin, alginate, heparin, hyaluronic acid, chitosan, agarose, etc., and/ or synthetics, such as polyethylene glycol (PEG), in association with synthetic polyesters like PLA, PGA, PLGA, PCL, or poloxamers [105,106,107,108].

In addition to scaffold techniques, other scaffoldfree techniques have also been proposed for TE in dentistry, such as [109]:

- pulp rebuilding with cell aggregates or pellets obtained by one-step centrifugation [110] or by more recent and precise methods of self-assembling [111];
- periodontal tissue engineering, like “cell sheet technology”, which consists in a non-invasive approach, using a thermo responsive polymeric material, named poly N-isopropyacrylamide (PIPAAm). A continuous monolayer of cells and ECM components (plated on PIPAAm surface) can be obtained with a slight decrease of temperature [112,113].

It has been proposed to change the cell sheet structure to a more flexible 3D pellet system—cell sheet-derived pellet (CSDP)—and pulp has been obtained by regeneration in a root canal after ectopic transplantation into immunodeficient mice models [114].

## 6. Nanostructured Materials in Use for Tissue Engineering in Dentistry

After the discussion of introductory aspects, we can say that the materials obtained using nanotechnology and currently used in TE in dentistry include a wide range of products ranging from simple nanopowders, to nanocarriers, to the establishment of complex scaffolds of different composition and structure.

### 6.1. Nanoparticles that Offer to the Tissue the Use of Their Chemical Components and Their Bioactivity

Calciophosphatic particles are often added in the simple form of filling nanopowders or carried by hydrogel in the bone healing sites, with other components, in order to promote remineralization [115]. In particular, the nanostructured hydroxyapatite (nano-HA) and nanoCaP nanomaterials have received considerable attention in the past decade [116,117]. The nano-HA has shown excellent biological performances compared to conventional HA [118,119,120,121]. Furthermore, nano-HA showed biocompatibility and bioactivity in respect of bone components, probably as a result of its similarity with the chemical component and mineral structure of bone tissue [97].

Moreover, due to their small size and large specific surface, nano-HAs may not only promote ion exchange within a physiological environment, but also increase protein absorption and cellular response [97], especially if stressed by physical means [122].

### 6.2. Nanostructured Materials for Drug or Signaling Molecules Delivery

These are nanomaterials that incorporate molecules which, once released, enhance the regenerative capacities of the tissues. They are often useful in TE.

They are bioactive nanocarriers represented by nanospheres, nanotubes, and nanofibers. Nanospheres are material-encapsulating polymer matrices used for slow and prolonged release of signaling molecules or drugs [90,123]: low-molecular-weight polymers form porous microspheres that release drugs slowly [90]. Electrospinning is considered to be the most viable methodology for the generation of scaffolds with varying compositions, according to the target tissues, and with a nanofibrous morphology [104]. Nanotubes, compared to nanosphere nanocarriers, provide larger inner volumes for filling desired chemicals or biochemical species and offers distinct inner and outer surfaces that can be differentially functionalized [124,125].

### 6.3. Nanostructured Materials for Build Scaffolds in Dentistry

Many methods have been developed to shape scaffolds for tissue-engineering applications and the conventional techniques include emulsion freeze-drying, phase separation, gel casting, precipitation, and solvent casting/salt leaching [104]. However, currently only three techniques can generate the nanoscale features suitable to nanoscaffold training: electrospinning, self-assembly, and phase separation [126].

The process of electrospinning consists of applying a strong electric field at the jet coming out from a syringe containing a solvent and the polymer interested. The electric field, creating a greater force than the superficial tension of the solution, gives a spiral shape and elongates the spray jet and, after solvent evaporation, only one set of polymer nanofibers remains. Nanofibers are deposited in a gatherer which can direct them and then solidify into a non-woven fabric that resembles cotton [126]. Electrospinning can be used for many synthetic and natural polymers and supports fabrication of nanofibers from synthetic and natural copolymers or polymer composites with biological molecules [127]. However electrospinning often cannot produce true nanofibers and cannot produce complex 3D scaffolds or designed pore geometry.

Self-assembly is the spontaneous interaction of components into a larger functional structure. This process occurs naturally (e.g., self-assembly of nucleic acids) and can be mimicked to form nanofibrous polymer scaffolds from engineered self-assembling peptides [128,129]. Molecular self-assembly often results in hydrogel formation containing true nanofibers [128]. They can also be used in injection applications in vivo, because the process can occur after injection. However, self-assembled hydrogels can have poor mechanical strength and are susceptible to uncontrolled enzymatic degradation [104,126].

Phase separation is a process where a single-phase homogenous polymer solution is solicited to the point of causing separation into a polymer-rich phase and a solvent-rich phase. This separation occurs to lower the system free energy due to the thermodynamically unstable state of the solution [130,131].

## 7. Experimental Studies in Dentistry with the Use of Nanomaterials

Although currently there are a limited number of studies where simple or complex nanomaterials have been used for tissue engineering in dental applications, they show excellent prospects in supporting the regeneration of enamel, pulpodentin complex, periodontal apparatus and teeth.

### 7.1. Enamel

Among the experimental studies that have brought to enamel regeneration it is necessary to distinguish [132,133] those that, with chemical-physical means, have brought to the precipitation of HA crystals in form to prism-like [134,135,136] from those that have used nanomaterials and TE techniques. In particular, nanofibres with RGD epitope sequence as signalling function on their surfaces have been used to facilitate the attachment, proliferation and differentiation of ameloblast-like cells [137] also in presence of signaling molecules [138]. Actually the results are partial, given the enamel peculiarities.

### 7.2. Pulpodentinal Complex

In the context of TE applied to the pulpodentinal complex regeneration, basing on current knowledge of the pulpal and dentinal biology [139], two paths were followed: the cell transplantation and the cell homing [80,81]. In the cell transplantation, the stem cells are isolated, cultivated in vitro, increased in number, inserted in soft scaffolds or hydrogels with or without addition of signaling molecules and calcium-phosphate nanoparticles and at last implanted in the receiving site, the empty and sterile endodontic space [140,141,142,143,144,145,146,147,148,149,150,151,152]. Instead, in the cell homing, where it is not necessary to isolate and manipulate in vitro stem cells, it is tried to reach pulpodentinal complex regeneration through the stimulation of residual stem cells by means of molecules veicolate by hydrogels [153,154,155].

Recently in the techniques there was the introduction of core-shell delivery sistems that can be produced like nanofibers, nanospheres and 3D assembled/constructed scaffolds of these and are obtained by means of co-concentric nozzle extrusion, microfluidics generation or chemical confinement reactions [109]. About these last techniques, Kim et al. in their review concluded that “recent work using novel biomaterials scaffolds and growth factors that orchestrate the homing of host endogenous cells represents a departure from traditional cell transplantation approaches and may accelerate clinical transplation” [156].

### 7.3. Periodontal Apparatus

In the medical and dental fields, the periodontics was one of the first that has made the attempt to repair and regenerate the structure and function of damaged tissues, through the use of barriers able to drive the exact regeneration of hard and soft tissue of periodontal apparatus, avoiding the colonization of the site damaged by unsuitable tissues (epithelial/connective) [34,157]. These membranes have first been not absorbable [32,158,159] and then adsorbable [33,160,161,162], bringing to two types of clinical application, the Guided Tissue Regeneration (GTR) and the Guided Bone Regeneration (GBR). It is one of the most fertile fields of applied clinical research and the recent introduction of the use of nanomaterials [78,163] has led to further advances: on the one hand, the use of nanopowders [164] but above all the development of periodontal membranes, both for GTR and GBR, obtained by nanotechnological methods such as film-casting [165,166,167], dynamic filtration [168] or electrospinnig [169,170].

Very interesting are the functionally graded periodontal membranes (FGMs) obtained by electrospinning: among them it is important to remember the electrospun PLGA membranes [171,172], the electrospun gelatin membranes [173] and the scaffolds, single layered PLLA/MWNTs (multi-walled carbon nanotubes)/HA membranes [174].

The evolution of nanotechnology then allowed to structure membranes with different layers like those obtained with sequential multilayer electrospinning; they consist of a core layer and two functional surfaces in contact with the bone (nanoHA) and the epithelium (metronidazole, MET); the core layer is obtained by a central portion of PLCL (polyactidecaprolactone) and by two layers of PLC/PLA hydrogels [175]. Another example of the degree of precision achieved in the membranes nanotechnology elaboration is biphasic scaffold, made of a Fused Deposition Modelling (FDM) for bone compartment and an alectrospun micro-fibrous membrane for periodontal ligament, intended to simultaneous delivery of the two cell types (PLC and osteoblasts) allowing the simultaneous regeneration of alveolar bone/periodontal ligament complex [176].

### 7.4. Entire Tooth

Over time, various techniques in the entire-tooth regeneration field were attempted [177,178], like the assembly of bioengineered component parts, the pellet engineering, the chimeric tooth engineering, the gene manipulated tooth regeneration [140,179,180,181]. Currently, the two most followed routes for the regeneration of the entire tooth can be identified in: scaffold-based tooth regeneration and simulation of the embryonic development of natural teeth [104,182].

The first method is to implant in vivo a scaffold with stem cells formed in vitro. At the beginning the technique consisted in inserting cells from swine dental germs in a scaffold then implanted into rats and it gave encouraging results (in about 15% of cases), but with training of dental structures smaller than normal teeth [183,184]. Then, there have been remarkable results using autotransplantation in the swine, getting tooth regeneration using dental bud cells alone or combined with bone marrow fluid in gelatin-chondroitin-hyaluronan tri-copolymer scaffold [185,186]. More recently, with the introduction of nanotechniques nanofibrous scaffolds based on PLLA/MWNTs/HA [187], PLLA/HA [188] or PCL/gelatin with or without HA [189] all obtained by electrospinning were used and the results were better though not decisive.

The second method is based on mimicking the embryological development to create natural teeth in animals using embryonic, neural and bone-marrow-derived stem cells without the use of a scaffold [190,191,192]. Perfect formed teeth were obtained then successfully implanted [193,194].

In both cases some doubts still remain about the applicability in humans and the clinical feasibility. In particular “remain to be determined whether such bioengineered teeth can achieve the masticatory function, biomechanical cooperation and sensory response of their naturally formed counterparts” [182].

## 8. Summary

The tissue engineering of dental oral tissue is facing significant changes in clinical treatments in dentistry. Different studies are present in the literature about nanomaterials in dentistry, all focused on the development and analysis of hard scaffolds, soft scaffolds (hydrogel), and nano-powders. All of these studies are animal trials and only a few of them are about human patients. This review shows a context of literature studies about nanomaterials in dentistry and their real applications in dental fields. This paper starts with a micro and macro morphological description of dental tissues, with a focus on the main diseases that may affect them and that can be clinically resolved with tissue engineering methods in the future. Stem cells in dental and periodontal tissues are usable in dentistry, and can be classified as pluripotent cells (for regeneration of dental tissues), mesenchymal cells, and epithelial cells. Growth factors and signaling molecules in some studies are focused on periodontal regeneration and less on pulpodentinal complex.

Some consideration can be given regarding the main biomaterials used to build scaffolds. In particular, it is important to distinguish rigid scaffolds, usable in bone tissues, from soft and injectable scaffolds for pulpodentinal and periodontal complex. The rigid biomaterial scaffolds used can be classified as natural and synthetic ceramics, polymers, and composites. For soft scaffolds, both natural (collagen, fibrin, alginate) and synthetic (PEG, PLA, etc.) materials can be used.

The review also reports the nanostructured materials for tissue engineering in dentistry, distinct from nanoparticles used for their chemical components and their bioactivity (calcium phosphate nanoparticles, nano hydroxyapatite), nanomaterials for drug or signaling molecules delivery (nanospheres, nanotubes, and nanofibers), and nanomaterials for scaffolds produced by three techniques (electrospinning, self-assembly, and phase separation). Table 2 summarizes the main nanomaterials used in dentistry, along with the applications and the studies reporting their production and use.

In conclusion, these materials are very important in bio-engineering and they can represent the vehicle for stem cells and/or growth factors in oral tissues for their regeneration.

## 9. Conclusions

There is no doubt that the development of nanotechnology offers exciting perspectives to regenerative dentistry in the near future. The combinatorial use of various stem cells, signaling molecules, and nanostructures (with creation of specific rigid or not rigid scaffolds) has already been obtained, in animals, for the regeneration of damaged dental tissues. However, there are still several problems concerning the safety and standardization of techniques that must be solved in the near future before clinical application in humans.

## Figures and Tables

**Table 1:** Comparative compositions and structural parameters of inorganic phases of enamel, dentin, bone, and hydroxyapatite.

Composition	Enamel	Dentin	Bone	Hidroxyapatite
Calcium (wt. %)	36.5	35.1	34.8	39.6
Phosphorus (wt. %)	17.7	16.9	15.2	18.5
Ca/P (molar ratio)	1.63	1.61	1.71	1.67
Carbonate (CO_3_^2−^) (wt. %)	3.5	5.6	7.4	-
Sodium (wt. %)	0.5	0.6	0.9	-
Magnesium (wt. %)	0.44	1.23	0.72	-
Potassium (wt. %)	0.08	0.05	0.03	-
Fluoride (wt. %)	0.01	0.06	0.03	-
Chloride (wt. %)	0.30	0.01	0.13	-
Pyrophosphate (P_2_O_7_^4−^) (wt. %)	0.022	0.1	0.07	-
Totale inorganic (wt. %)	97	70	65	100
Total organic (wt. %)	1.5	20	25	-
Water	1.5	10	10	-
a axis (nm)	0.9441	0.9421	0.941	0.9430
c axis (nm)	0.6880	0.6887	0.689	0.6891
Cristallinity Index (HA = 100)	70–75	33–37	33–37	100
Cristalline size (nm)	100 × 90 × 30	35 × 25 × 4	50 × 25 × 4	200–600
Ignition products (800 °C)	β-TCP + HA	β-TCP + HA	HA + CaO	HA
Elasticity modulus (GPa)	80	15	0.34–13.8	10
Compressive strenght (MPa)	10	100	150	100

**Table 2:** Summary of various nanomaterials used for tissue engineering in dentistry.

Nanomaterials	Applications	References
silver and zinc oxide nanoparticles	toothpastes, mouthwashes and composite resins for prevention of caries and periodontal diseases (antibacterial and antidemineralizing properties)	[10,11,13]
amorphous calcium phosphate nanoparticles		[12]
carbonate hydroxyapatite nanocrystal		[14]
calcium carbonate nanoparticles		[15]
calcium phosphate nanoparticles	toothpastes, composite resins and dental adhesives for remineralization of tooth lesions	[16,17]
gold nanoparticles	diagnosis of malignant and pre-malignant oral diseases	[18,19,20]
semi-conductor nanocrystals		[21]
nano-textured surfaces	surface modifications of dental implants	[25,26,27,28,29,30,31]
nanostructured hydroxyapatite	promotion of bone remineralization	[97,116,117,119,120,121,122]
carbon nanotubes	bone repair/regeneration	[125]
polymeric nanofibrous scaffold	dental and craniofacial applications	[126]
polycaprolactone nanofibers	scaffold for bone tissue engineering-response to osteogenic regulators	[127]
peptide-amphiphile nanofibers	scaffold for bone tissue repair	[128]
bioactive peptide -amphiphile nanofibers	enamel regeneration	[137,138]
nanohydroxyapatite	periodontal tissue repair and regeneration	[164]
nano-carbonated hydroxyapatite/collagen/PLGA membrane		[165,166]
nano hydroxyapatite/polyamide 66 GBR membrane		[167]
chitosan/nanohydroxyapatite composite membrane		[168]
polycaprolactone/calcium carbonate composite nanofibers membrane		[169]
nano-apatite/PCL composite membrane		[170]
poly(DL-lactide-coglycolide) nanofibrous membrane		[171]
gelatin nanofibrous membrane		[173]
PLLA/MWNT/HA membrane		[174]
PLLA/MWNTs/HA, PLLA/HA, PCL/gelatin/HA nanofibrous scaffolds	entire-tooth regeneration	[187,188,189]

## Abbreviations

The following abbreviations are used in this manuscript:

TE	Tissue Engineering
NM	Nanomaterials
NIR	Near-infrared
GNPs	gold nanoparticles
OFNASET	oral fluid nanosensor tests
CRP	C reactive protein
GTR	tissue guided regeneration
EDJ	Enamel-Dentin Junction
ERM	Epithelial Rests of Malassez
DEJ	dentin-enamel junction
HA	hydroxyapatite
PDL	periodontal ligament
DPSC	dental pulp stem cells
SCAP	Stem cells from apical papilla
DFSC	dental follicle stem cells
BFGF	basic Fibroblast growth factor
PDGF	Platelet derived growth factor
SCF	stem cells factor
G-CSF	Granulocyte colony-stimulating factor
CaP	calcium phosphate
ACP	amorphous CaP
PLA	Polyactic acid
PGA	Polyglycolic acid
PLGA	PolylactideCo-glycolide
PCL	Poly-caprolactone
PEG	polyethylene glycol
nano-HA	nanostructured hydroxyapatite
GTR	Guided Tissue Regeneration
GBR	Guided Bone Regeneration
FGMs	functionally graded periodontal membranes
MWNTs	multi-walled carbon nanotubes
MET	metronidazole
PDL	Periodontal ligament
PLCL	polyactidecaprolactone
FDM	Fused Deposition Modelling
